# Mary Ellen Avery’s Research Career – Remembrance of Things Past

**DOI:** 10.3389/fped.2014.00034

**Published:** 2014-04-28

**Authors:** John Steven Torday

**Affiliations:** ^1^Department of Pediatrics, University of California Los Angeles, Los Angeles, CA, USA

**Keywords:** Mary Ellen Avery, lung surfactant, respiratory distress syndrome, evidence-based medicine, leadership

## Abstract

Mary Ellen Avery’s research is recognized as a milestone in biomedical research. She had discovered the underlying cause of hyaline membrane disease, surfactant deficiency, fostering ever more vigorous efforts to reduce neonatal mortality in the burgeoning practice of Neonatology. Neonatology is the only clinical discipline that began as an experiment, making it a model for biomedical research. Avery knew that the concerted effort to treat preterm newborns could potentially do more harm than good, violating her oath to Hippocrates, if not held to the highest scientific standards. She remained true to that pledge throughout her career, as recounted in this Review.

## Introduction

I feel like the narrator recounting his tale at the wedding banquet in Coleridge’s epic poem “The Rime of the Ancient Mariner.” Of course anyone can go to PubMed and retrieve Mary Ellen Avery’s publications, totaling 146 peer-reviewed papers, but the back story is what I am going to relate, largely based on my personal recall under her tutelage for 25 years.

Her interest in the breathing problems of newborn infants had been piqued by the awareness that the most common finding in the lungs of premature infants born alive who died shortly thereafter, was atelectasis and hyaline membranes. The pathology had been well-described by both George Anderson and Peter Gruenwald at Johns Hopkins. They both emphasized the lack of a clinical description of the course of the disease. In 1947, Gruenwald (1) described the unusual expansion patterns of the lungs of premature infants. He hypothesized that an unusually high surface tension could account for the high pressure necessary to introduce air into the lungs, but also that air was trapped in the lungs in a Swiss cheese-like pattern, as predicted by the Law of LaPlace.

Richard Pattle (2) was studying the foam of pulmonary edema in the Chemical Defense Establishment in Porton, England, since some gases used in wartime such as phosgene induce lung edema, so antidotes were being sought. The unusual stability of bubbles expressed from normal lungs led Pattle to conclude that the internal surface of the lung must be covered with a lining layer of very low surface tension. He suggested that absence of the lining substance of the alveoli might play a role in causing atelectasis. The appearance of hyaline membranes might be due to a defective lining layer causing transudation from the blood, or to excessive secretion of the lining substance itself.

Dr. Avery had completed her pediatric residency at Johns Hopkins in the 1950s, so she went off to Boston to study respiratory physiology at the Harvard School of Public Health with Jere Mead, in conjunction with a Fellowship to study newborn infants with Clement Smith at the Boston Lying-In Hospital.

Mead’s laboratory had discovered that if the lung was filled with air it had greater elastic recoil than if it were filled with saline, leading to the realization that the surface forces of the lung, which are greater at an air–liquid interface than at a liquid–liquid interface, caused the elastic recoil of the lung. Mead’s group used these observations to calculate lung surface area, which differed substantially from that estimated by the morphologists. That observation prompted Clements to measure the surface tension of material expressed from the lung. Clements, tried to reconcile the Mead laboratory data with Pattle’s findings of stable bubbles expressed from lungs having zero surface tension. Whittenberger at the Harvard School of Public Health, a research advisor to Clements at Edgewood Arsenal, Maryland communicated Clements’ findings to Mead and Avery back in Boston. Clements had reasoned that a dynamic method of measurement of surface tension would better reflect conditions in the lung, so he designed a modified Wilhelmy surface film balance to study changes in surface tension with area. His striking observation established the important feature of the alveolar lining layer, namely a change in surface tension with area, so that at large lung volumes surface tension is high, and at low lung volumes it approaches zero. He named the material presumed to be at the alveolar–air interface “pulmonary surfactant,” and commented on its central role as an anti-atelectatic factor.

Avery visited Clements’ lab at the Edgewood Arsenal in December, 1957 to see the surface film balance. On her return to Boston, Mead proposed a way to modify the method to allow them to study minced extracts from lungs of human infants. Samples of lungs were obtained courtesy of Kurt Benirshke, the chief of pathology at the Boston Lying-In Hospital at that time. The absence of foam in the lungs at autopsy was a prominent observation that might have led to the conclusion that these lungs were deficient in surfactant even in the absence of measurements on the surface film balance. The first measurements were made before Pattle had published his observations in 1958. His finding that the bubbles expressed from lungs of immature guinea pigs were unstable reassured Avery that she was on the right track in her studies of the lungs of infants who had died of hyaline membrane disease (HMD).

There were multiple theories for the pathogenesis of HMD when Avery began her study of lung surfactant. In the first edition of her book *The Lung and its Disorders in the Newborn Infant* (3), she objectively presented what was known at the time, and, although she presented observations on the possible role of surface forces, she admitted that the etiology of HMD was unknown. She recapped the arguments for the primacy of aspiration, asphyxia, heart failure, shock, disturbed autonomic regulation, fibrinolytic enzyme defect, prolonged acid–base derangements, and low serum proteins. Over the ensuing years these variables have been eliminated one by one, bringing ever-greater clarity to the ultimate role of surfactant deficiency. The first time Avery unequivocally stated that HMD was due to surfactant deficiency was in the fourth edition of her textbook in 1981.

## Respiratory Distress Syndrome as Surfactant Deficiency – Evidence-Based Medicine

The first four peer-reviewed papers Avery published were case reports, beginning in 1955 (4). But then there was that watershed year of 1959 when she and Jere Mead published their ground-breaking paper on HMD as surfactant deficiency (5). She would tell her students how difficult it was to publish this manuscript because it went against convention – Hochheim had declared that HMD was an obstructive disease due to the eosinophilic membranes found in the airways of the newborns who had died of this disease. But Avery was aware of the studies done by Von Neergaard and Pattle, showing that there was surface tension reducing activity in the alveoli of the mammalian lung. She reasoned that if these infants were surfactant deficient that that would have accounted for the atelectasis and exudation of fluid across the alveolar wall, producing the hyaline membranes. If she was right, there was an opportunity to correct the disease, in contrast to the assumed intrauterine obstructive mechanism associated with HMD. From that point forward Dr. Avery published another 141 papers, but of those there were 26 that would plot her arc as the clinician-scientist who conquered HMD. I would like to recount those studies within the context of Dr. Avery’s effort to validate HMD as Respiratory Distress Syndrome, or surfactant deficiency disease. In early studies excised lungs of human newborns were used (6) in tandem with animal models to establish the relationship between surfactant and lung function (7), and the expression of lamellar bodies in alveolar type II cells as a function of development (8). And since the functional surfactant was predicated on its secretion by the alveolar type II cell, an elegant histologic study was published demonstrating this property of the alveolar epithelium (9). In a series of follow-up studies, Avery and her colleagues demonstrated relationships between conventional knowledge of pulmonary alveolar homeostasis and lung surfactant at the cellular, functional, and pathophysiologic levels (10–15) to further convince the scientific community of the mechanistic relevance of the surfactant system to alveolar homeostasis. Subsequent studies were designed to try and identify factors that might accelerate the appearance and activity of surfactant in order to prevent RDS (15), including observations that hormonal acceleration of lung maturation was physiologic in nature (16). Such studies were done in conjunction with the further elucidation of those factors that merely caused respiratory distress, such as edema (17) and retained fetal lung fluid (18), versus those that specifically caused RDS as surfactant deficiency disease, strictly defined as dependence on oxygen support in association with grunting, flaring, and retracting of the thorax, and a ground-glass appearance of the lung on x-ray examination.

The breakthrough in the treatment of surfactant deficiency *in utero* came when Liggins discovered that antenatal glucocorticoids could accelerate lung maturation. Avery’s laboratory performed a systematic series of studies to demonstrate the physiologic effect of glucocorticoids on lung surfactant production in both rabbits (19–23) and lambs (24), including untoward effects like the inhibition of lung growth (25) for “full disclosure” – Avery wanted her colleagues to be totally informed about this emerging therapy. Subsequent studies filled in gaps in the relationships between physiologic and pathophysiologic agents and surfactant dynamics (26, 27) so as to further elaborate on the basic and clinical aspects of surfactant biology for the scientific community. Among these was the study by Wyszogrodski (28) showing that beta adrenergic agents caused surfactant secretion, an important observation for both basic and clinical understanding of surfactant’s properties. The last scientific peer-reviewed paper that was co-authored by Dr. Avery was the demonstration of the sexual dimorphism in the rate of lung maturation during human fetal development (29), capping a series of animal studies conducted in my laboratory with Heber C. Nielsen. Those studies were designed to determine why males were not as responsive to antenatal glucocorticoids as females, an observation first reported by Kotas and Avery (30).

**Figure 1:**
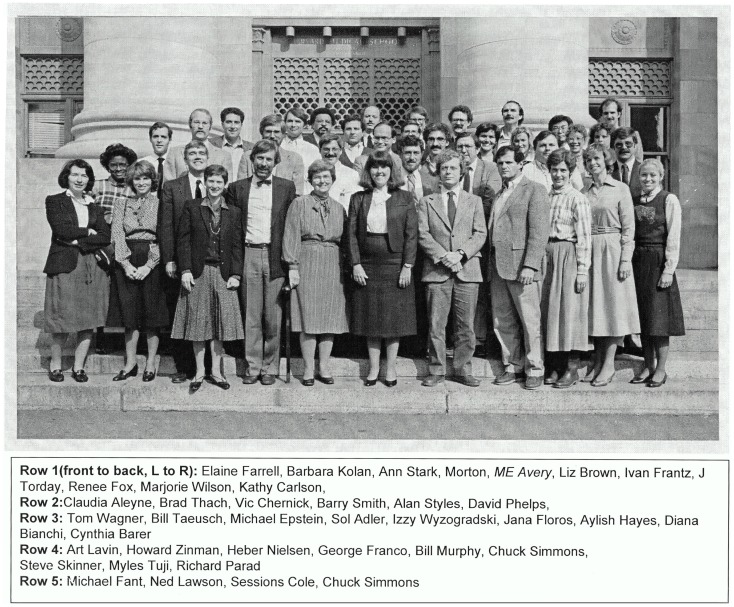
**The Joint Program in Neonatology (JPN), 1984, in front of the Administration Building (Building C), Harvard Medical School**. In 1984, on the 25th anniversary of the publication of the Avery and Mead paper, members of the Joint Program in Neonatology, which Dr. Avery had created in 1974, gathered for a group picture with her in front of the administration building at Harvard Medical School. Those in attendance were clinicians, clinician-scientists, and basic scientists alike as the embodiment of Dr. Avery’s eternal effort “to do no harm.”

In 1984, on the 25th anniversary of the publication of the Avery and Mead paper, members of the Joint Program in Neonatology, which Dr. Avery had created in 1974, gathered for a group picture with her in front of the Administration Building at Harvard Medical School (see Figure 1). Those in attendance were clinicians, clinician-scientists, and basic scientists alike as the embodiment of Dr. Avery’s eternal effort “to do no harm.”

In addition to her research efforts, Dr. Avery was a champion for women in the field of medicine. Along with Lynn Reid and Mary Ellen Wohl, she strongly advocated for a level playing field as the first woman ever to have become the Chair of a clinical department at Harvard Medical School. When she was applying to medical school she was rejected by Harvard Medical School because of their policy of excluding women – always the student of history, fighting the hard fight for what was right despite the prevailing attitudes, whether in science or social justice.

Dr. Avery’s last cited paper, entitled “What is good for children is good for mankind: the role of imagination in discovery” was her formal Address to the American Association for the Advancement of Science as the President of the society in the year 2004 (31). In her speech, she emphasized the power of the imagination to overcome mankind’s problems. She certainly demonstrated her own ability to achieve that goal, and even surpassed it through her leadership and mentoring.

I can only hope that her spirit will marshal on.

## Conflict of Interest Statement

The author declares that the research was conducted in the absence of any commercial or financial relationships that could be construed as a potential conflict of interest.
